# Supplementary material for: Childhood Maltreatment and Adult Work Absenteeism: Work Meaningfulness as a Double-Edged Sword
Source: Int J Environ Res Public Health. 2024 Apr 7;21(4):451. doi: 10.3390/ijerph21040451 (PMC11050126; doi:10.3390/ijerph21040451)
Supplement: Supplementary file 1 [file ijerph-21-00451-s001.zip › ijerph-2859396-supplementary.pdf]

Child maltreatment and adult work absenteeism: work meaningfulness as a double-edged sword

Table S1 - Demographic Characteristics of Participants

	Maltreated employees		Non-maltreated employees		Full sample	
	n	%	*n*	%	*n*	%
**Gender**						
Female	107	93%	185	88.9%	292	90.4%
Male	8	7%	23	11.1%	31	9.6%
**Marital Status**						
Single	21	18.4%	41	19.7%	62	19.2%
Married/partnered	82	71.9%	154	74%	236	73.1%
Divorced/separated	8	7%	12	5.8%	20	6.2%
Widowed	3	2.6%	1	.5%	4	1.2%
Missing	-	-	-	-	1	0.3%
**County of Birth**						
Israel	98	85.2%	184	88.5%	282	87.3%
Former Soviet Union	8	7%	11	5.3%	19	5.9%
Ethiopia	3	2.6%	4	1.9%	7	2.2%
Other	6	5.2%	9	4.3%	15	4.6%
**Religion**						
Judaism	104	90.4%	187	90.3%	291	90.1%
Christianity	1	0.9%	6	2.9%	7	2.2%
Islam	9	7.8%	12	5.8%	21	6.5%
Other	1	0.9%	2	1%	3	0.9%
Missing	-	-	-	-	1	0.3%
**Income**						
Below average	70	63.1%	124	59.3%	195	61.5%
Average	22	19.8%	50	23.9%	68	21.5%
Above average	19	17.1%	35	16.8%	54	17%
Missing	-	-	-	-	6	1.9%
**Managerial position**						
Yes	25	22.1%	46	22.9%	71	22.6%
No	88	77.9%	155	77.1%	243	77.4%
Missing					9	-

Table S2 - Demographic and Work-related Characteristics of Participants

	Maltreated employees		Non-maltreated employees		Full sample		Sample size *(N)*
	Mean	*SD*	*Mean*	*SD*	*Mean*	*SD*	
**Age**	39.35	10.45	40.07	10.13	39.82	10.24	314
**Years of Education**	16.78	2.58	17.03	2.20	16.94	2.34	317
**Working hours (per week)**	36.53	9.07	37.38	11.28	37.08	10.54	310
**Years at current role**	5.74	6.7	7.14	7.81	6.64	7.54	284
**Years at current organization**	6.34	7.33	7.11	7.51	6.84	7.45	267

Figure S1 – Frequency of absent days from work (last 3 months)

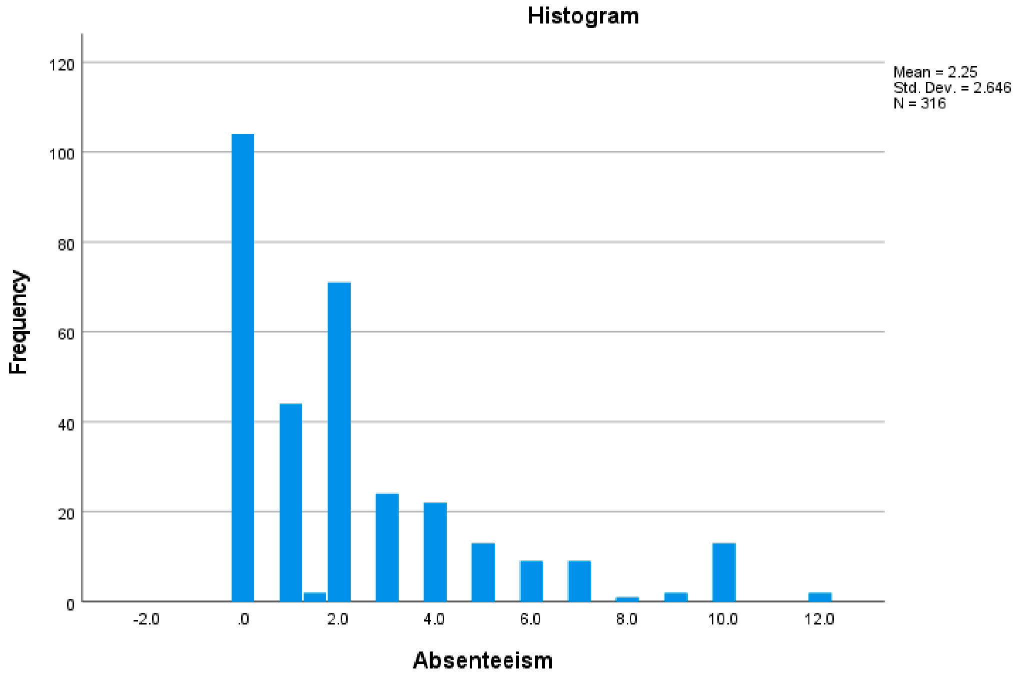

Table S3 - Prevalence of Childhood Exposure to Abuse and Neglect by Category (Full Sample - Maltreated and Non-maltreated Employees)

Category of maltreatment childhood exposure	Prevalence (%)
**Abuse by category**	
Psychological	22.0%
Physical	13.4%
Sexual	11.8%
**Neglect by category**	
Emotional	15.6%
Physical	3.1%
**Witnessing domestic violence**	5.4%
**Any category reported**	34.7%
